# What Drives Health-Care Spending Priorities? An International Survey of Health-Care Professionals

**DOI:** 10.1371/journal.pmed.0040094

**Published:** 2007-02-20

**Authors:** Glenn Salkeld, David Henry, Suzanne Hill, Danielle Lang, Nick Freemantle, Jefferson D'Assunção

## Abstract

The authors set out to compare spending priorities for health care, across a selection of largely middle-income countries, through a survey of current and future decision makers.

## Introduction

Making the rules of health-care resource allocation transparent is a challenge for all governments. The Oregon Health Plan in the late 1980s was one such attempt to prioritise expenditure of limited Medicaid funds, based on public values [1]. For decision makers, asking the general public and health professionals to express their preferences for health-care spending priorities can be a way of ensuring that the process and resultant spending priorities are seen as legitimate and fair [2]. In a study comparing the preferences of health professionals and members of the public for setting health-care priorities, Wiseman found considerable uniformity in preferences between the two groups [2]. However, some members of the public argued that it would be better to trust health professionals to make the correct decision in the first place.

Those entrusted to set health-care priorities do so according to what is in the best interest of the public. This in turn requires those decision makers to make value judgments on what constitutes “good”. On what basis should one health program deserve a higher priority for funding than another? Several studies have found that the general public and health professionals may not agree on who and what is most deserving of scarce health resources.

Based on an opinion poll, Groves showed that the public strongly disagreed with doctors and health managers on where best to spend health resources [3]. Myllykangas and colleagues, in a study on attitudes to health-care priorities, found that doctors and nurses were less inclined to be punitive towards funding for patients with self-induced diseases than the general public [4]. Yet Dolan et al. found that when the public were given time to listen to the considered opinions of their fellow citizens and reflect on their views, fewer were willing to discriminate against people with what might be regarded as self-induced diseases [5].

In all cases it is values, the building blocks or rules which govern attitudes and behaviour, that are reflected in priorities for spending in health care [6,7]. The values of the decision makers clearly count in setting health-care expenditure priorities. So do decision makers themselves share common values about priorities for health-care spending? Are there any similarities in values between decision makers in different countries?

The purpose of this study is to compare spending priorities for health care across a selection of predominantly middle-income countries, based on the opinions of current and future decision makers. Using an opinion poll questionnaire, we surveyed 253 health professionals from six countries, asking them to rank ten health interventions in order of priority for spending from most important (rank 1) to least important (rank 10). The questionnaire was based on a short questionnaire on priorities for health-care spending developed by Groves [3].

**Box 1. Median Rankings of Health-Care Spending Priorities Across All Countries, in Order of Importance**

1. Childhood immunisation
2. Anti-smoking education for children
3. GP care for everyday illness
4. Screening for breast cancer
5. Intensive care for neonates
6. Support for carers of the elderly
7. Treatment for people with schizophrenia
8. Hip replacement
9. Heart transplant
10. Cancer treatment for smokers

The questionnaire asked respondents to imagine that they were responsible for health-care spending in their country. This was followed by a question on whether or not they thought that funding for health care should be unlimited. No additional information was given to respondents. The survey was designed as an introductory learning exercise for a series of intensive workshops (of three to ten days' duration), run under the auspices of the World Health Organization or AusAID, the Australian government's overseas aid program (South Africa workshop only), on the application of evidence-based medicine and economic evaluation to the selection and reimbursement of pharmaceuticals. The intention was to introduce course participants to the notion of priority setting. The questionnaire was administered at the beginning of each workshop. Details of the study setting and participants, questions used to prompt group discussion, and the data analysis are outlined in Text S1.

## Spending Priorities

**Figure 1:**
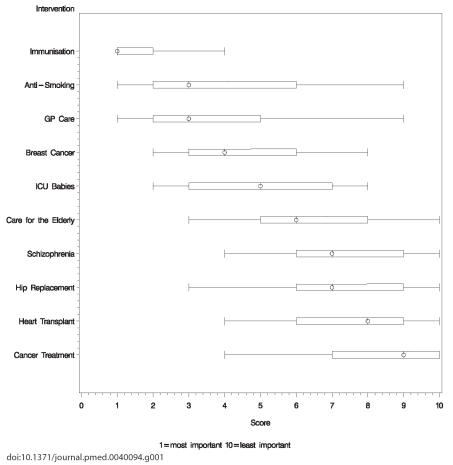
Spending Priority: Intervention

A summary of the intervention rankings, pooled across countries, is shown in Box 1. Across all countries, childhood immunisation was the highest ranked intervention and cancer treatment for smokers was ranked as the least important priority for health-care spending (Box 1). There was little variation across countries in the median rank score for preventive health care and greatest variation for “lifesaving” interventions (Figure 1). The Kruskal-Wallis test for the null (that the median ranks were equal across countries) could not be rejected at the 5% significance level for the following interventions: childhood immunisation (*p* = 0.114), antismoking education for children (*p* = 0.327), screening for breast cancer (*p* = 0.355) and treatment for people with schizophrenia (*p* = 0.317). For all other interventions the null hypothesis was rejected at the 5% level, suggesting that the median ranks for these interventions are significantly different across countries. The Kruskal-Wallis test results did not change at the 5% significance level for the all country sample that excluded the South African pharmaceutical industry respondents.

**Figure 2:**
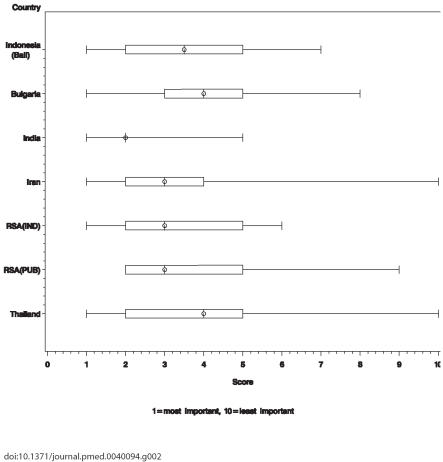
Spending Priority: GP Care

Primary care (by a general practitioner [GP]) was ranked highest by participants in India (rank 2), Iran (rank 3), and public sector and industry participants in South Africa (rank 3) (see Figure 2). Conversely, heart transplant was ranked lowest in Iran (rank 8) and India (rank 9). The life-saving intervention of neonatal intensive care was ranked highest by participants in Bulgaria (rank 3) and lowest by those in India and Iran (rank 5 and 6, respectively). Most respondents thought that funding for health care should not be unlimited, ranging from 68% in Bali (Indonesia) to 90% in South Africa.

## Key Values

### Prevention.

The strongest and most consistently shared value across countries was a general preference for preventive health care over curative care. When asked to state their criteria for ranking interventions, participants regarded childhood vaccination as safe, affordable, efficacious, and cost effective. Anti-smoking education for children was seen in the same light as immunisation and breast cancer screening was regarded as a worthwhile and cost-effective intervention. This strong and consistent preference for prevention over cure is quite at odds with the actual spending priorities in most countries throughout the world. In 2004, OECD (Organisation for Economic Co-operation and Development) member countries spent on average only 2.8% of total health expenditure on organised public and private prevention programmes [8]. Reliable data on the proportion of total health expenditure spent on prevention and public health for the countries in this study are not available.

### Individual responsibility.

Treatment for schizophrenia elicited the greatest variation in rankings within countries but a consistent (and statistically significant) low median ranking between countries. When discussing the reasons for this ranking, participants admitted that mental illness was stigmatised in their country and that this was reflected more generally in the low levels of funding for mental health. The visual presentation to the large group of the median ranking for treatment of schizophrenia along with the 5th and 95th percentile prompted some discussion about whether the reasons for the low ranking were acceptable or not. Often respondents who ranked the intervention as a higher spending priority would state their reasons (for example, the existence of known cost-effective pharmacological therapies), but few people expressed a desire to change their ranking. Rather there was an acceptance that variation in rankings existed within the group. Likewise there was an acceptance of the rankings for the lowest ranked intervention, cancer treatment for smokers. Here, participants seemed to invoke the principle of individual responsibility. Smokers were “blamed” for their cancer and were regarded as the least deserving of health-care spending. This belief may have been tempered by the perception that treatment for lung cancer may not produce much health gain and hence may not be cost effective.

In a study on the effect of discussion and deliberation on the public's view of priority setting in health care, Dolan et al. found that while 57% of their lay public sample stated that smokers should have a lower priority for treatment compared to other groups on initial survey, after deliberation, only 37% gave smokers a lower priority as a final response [5]. In this study the authors found the respondents less willing to assign personal responsibility after some reflection and discussion.

### Fair innings.

At the top (most important priority for spending), participants favoured giving priority in spending to children. This is consistent with other studies that have found that policy makers give priority to interventions which target the young [9]. Newborns and infants were considered to be entitled to a fair start in life within certain limits. Those limits were defined by affordability and effectiveness. Neonatal intensive care was regarded as an expensive technology with variable health outcomes but participants in many countries apparently felt it was important that equity should override efficiency concerns when dealing with the life of a newborn. Just as Nord and colleagues found that people derive a benefit from the knowledge that society is “just” [10], respondents in our survey considered “fairness” important when ranking the interventions.

In contrast, interventions such as hip replacement and caregiver support, where the primary beneficiaries were older people, were regarded as a lower priority for health-care spending. The notable exception was Iran (a country with a young population) where participants ranked caregiver support midway (rank 5). For countries other than Iran, it may be that survey respondents adopted the “fair innings” principle whereby someone who has already had a fair innings, say a fit elderly person, gets lower priority for health-care spending than a young person who, “without treatment, will certainly not reach the societal norm (through premature death and/or lifelong disability)” [11]. What's not obvious from the results is the degree to which participants regard reducing health inequality as more important than achieving a health maximisation objective.

### Rule of rescue.

Participants were willing to invoke the “rule of rescue” [12]—the moral imperative to save the life of an identified individual who would otherwise die—but only up to a point. Whilst the survey was not designed to identify any rule of rescue threshold, individual participants said that they considered the health outcomes first and then cost as part of their decision-making criteria.

The median rankings of interventions did not differ between the South African pharmaceutical industry participants and the pooled results for the public sector participants in all countries. Further studies are needed to test whether the agreement in values between the industry and public sector respondents on some of the underlying principles for public sector resource allocation are reproducible in other countries.

### Opinion polls.

When asked what additional information they would have liked, participants wanted information on the benefits, harms, and costs of the intervention. Less often, participants identified the issue of scale—that is, how much more (or less) of something should be done. It is rarely the case that the decision to spend money on an intervention is dichotomous (yes/no); decisions are more likely to turn on how much should be spent. This in turn led some participants to conclude correctly that opinion polls cannot address the question of opportunity cost (the benefits forgone in sacrificing spending on one intervention for another) or the margin (how much more or less of an intervention should be funded), in the absence of data on comparative efficacy, safety, cost-effectiveness, and affordability.

## Limitations of Our Approach

This survey was intended as an educational exercise to introduce workshop participants to the notion that priority setting in health care is a value-laden exercise and one that should be informed by evidence-based medicine and economics. The interventions used in the survey, replicated from the Groves study [3], are formulated in very general terms. For example, GP care for everyday illness covers a wide category of services, from preventive measures to curative services. This limits our ability to make strong conclusions about one type of intervention versus another. There is a risk of confounding in the results due to the method of selection of our sample. The study participants were self-selected; they chose to attend the course. To the extent that policy makers who attend courses are systematically different from those who do not, this may have affected the extent to which subjects are representative of a population of health decision makers.

## Do the Preferences of Experts Accord with Those of General Populations?

Whilst the results of this survey do not allow for a comparison between the preferences of health professionals and the general population, other studies have shown a reasonable level of uniformity of opinion, with a few exceptions. Wiseman found that the public gave equal weighting to health professionals for public health/prevention interventions but more weight (for spending) to coronary artery bypass grafting and less to hip replacement than did the health professionals [2]. But overall, there was considerable uniformity of preferences between the two groups. Similarly, Myllykangas et al. found that the views of health professionals, local politicians, and the general public were generally similar, although the views of doctors differed substantially on some matters [4]. On the other hand, Groves found that the public tended to put life-saving interventions such as heart transplants and intensive care for babies higher up the spending priority list than doctors or National Health Service managers (who themselves ranked life-improving treatment as twice as important as lifesaving ones) [3].

## Conclusion

The strongest opinions elicited from our sample of health professionals, a general preference for prevention and for spending on the young over the old, bear little semblance to how health care dollars are actually spent in many countries. Other opinions, such as a preference to rescue an identifiable life in danger and a tendency to assign blame for disease, seem to exert more influence over current health care spending. The values expressed here transcended national and sectoral boundaries. Across the world many countries are struggling with the health and financial implications of a rapid rise in non-communicable disease. If health care professionals and policy makers believe that prevention and targeting the young is an important principle for health spending priorities, then health care funders should examine the cost effectiveness evidence for intervening early in life. Whilst the “rule of rescue” will always be a significant influence in health-care spending priorities, greater attention needs to be given to those interventions that are life improving as well as life extending. 3

## Supporting Information

Text S1 Research Methodology (24 KB DOC).
