# Supplementary material for: What Drives Health-Care Spending Priorities? An International Survey of Health-Care Professionals
Source: PLoS Med. 2007 Feb 20;4(2):e94. doi: 10.1371/journal.pmed.0040094 (PMC1800312; doi:10.1371/journal.pmed.0040094)
Supplement: Supplementary file 1 [file pmed.0040094.sd001.doc]

**Supplementary Online File:**

**Box S1	Research Methodology**

***Survey setting and participants***

The study participants were self-selected; they chose to attend the course at the invitation of the regional WHO office and health ministry. Similarly, the workshops were conducted in countries at the invitation of WHO or AusAID and the relevant Ministry of Health. Workshops were conducted in the following countries in the period from 2001 to 2005: Bali (Indonesia) (n=22), Bulgaria (n=16), Iran (n=51), India (n=22), South Africa – Public Sector (n=21), South Africa – Pharmaceutical Industry (n=67) and Thailand (n=54) were all employed in the public health sector with some responsibility for the selection, reimbursement or distribution of prescribed medicines. The exception to this was the South African Pharmaceutical Industry participants, all of whom worked for pharmaceutical companies in South Africa. . Across all countries, most participants had a professional background in pharmacy and there was a fairly even mix of men and women in the groups. The participants in the Iran, Bulgaria and India courses were exclusively from the host countries. For the other courses, most participants were from either the host country or region.

***Group Feedback***

In each workshop, following individual completion of the opinion poll exercise, participants were asked to consider in small groups (6-8) the following questions:

- What were your criteria for ranking interventions?
- What additional information, if any, would you have liked?
- Are opinion polls a good way to set priorities for health care resource allocation?

This provided participants with an opportunity to reflect on their values prior to the presentation of the results to the whole group of the opinion poll they had just completed. Based on respondents’ verbal answers to the questions above a set of ‘values’ emerged that provides the context for the inter country comparison of results. The verbal answers were written on paper or board and the key values identified are based on a description of the content and most frequent responses by survey participants.

***Statistical Analysis***

A descriptive analysis of the ranked data for each country was performed. The results are presented as box plots where the inter-quartile range is shown by the length of the white box, the median rank is traced across the boxes by a line inside a circle, and the lower and upper whiskers show the 5^th^ and 95^th^ percentile rank respectively. To gauge the extent of shared rankings across countries, a Kruskal Wallis non-parametric test for equality in the median location of responses across countries, by intervention, was performed. Two respondents were excluded from the final data analysis, one from Thailand and one from Bulgaria. Both ranked cancer treatment for smokers as the most important spending priority and childhood immunisation as the least important. Their responses may indicate confusion with the rank anchor points (level of importance) or an unwillingness to adopt the position of health care decision maker as instructed in the survey. The results are reported for the pooled responses for all participants in all countries. The Kruskal Wallis non-parametric test was also calculated for all participants, excluding the South African Pharmaceutical Industry respondents, and compared to the test results for the whole (including pharmaceutical industry respondents).
